# Assessment of the Diagnostic Value of Novel Biomarkers in Adult Patients With Acute Appendicitis: A Cross-Sectional Study

**DOI:** 10.7759/cureus.32307

**Published:** 2022-12-07

**Authors:** Murat Duyan, Nafis Vural

**Affiliations:** 1 Emergency Medicine, Antalya Training and Research Hospital, Antalya, TUR; 2 Emergency Medicine, Akdeniz University Hospital, Antalya, TUR

**Keywords:** neutrophil-to-lymphocyte ratio, monocyte-to-lymphocyte ratio, systemic immune inflammation index, diagnosis, biomarker, acute appendicitis

## Abstract

Background

Acute appendicitis (AA) is one of the most frequent causes of abdominal pain requiring emergency intervention in adults. Approximately one-third of cases present with atypical clinical symptoms. This study aims to compare the monocyte-to-lymphocyte ratio (MLR), red cell distribution width (RDW) to lymphocyte ratio (RLR), and systemic immune inflammation index (SII) with other biomarkers in distinguishing patients with and without AA.

Methodology

A total of 347 patients (AA 125, nonspecific abdominal pain 90, and control group 132) were enrolled in the study according to the cross-sectional study design. Receiver operating characteristic (ROC) analysis was used to determine the cutoff in diagnostic value measurements. Statistical significance was determined by the statistics of sensitivity, specificity, positive predictive value, and negative predictive value. Comparison of ROC curves of C-reactive protein (CRP), white blood cell (WBC), neutrophil count (NEU), neutrophil-to-lymphocyte ratio (NLR), MLR, and SII was evaluated with the pairwise comparison of ROC curves and 95% confidence interval.

Results

In detecting AA, CRP, WBC, NEU, NLR, MLR, and SII have excellent diagnostic power (area under the curve [AUC] 0.80-0.88), while RDW, lymphocyte count, monocyte (MON) count, and RLR had acceptable diagnostic power (AUC 0.70-0.77). When the power in the diagnosis of AA was compared, a significant difference was found between CRP and NEU, CRP and SII, WBC and NEU, and WBC and SII.

Conclusions

The diagnosis of AA remains dependent on many factors. Inflammatory biomarkers assist this process. MLR and SII may be recommended to use in diagnosing AA in adults, along with other clinical findings. RLR is adequate but not superior.

## Introduction

Acute appendicitis (AA) is one of the most common causes of abdominal pain in adults that necessitates immediate medical attention [1]. Approximately one-third of cases have atypical clinical symptoms [2]. It is frequently misdiagnosed due to the wide range of abdominal pains that require and do not require surgery. Various inflammatory and infectious conditions in the right lower quadrant may mimic the signs and symptoms of AA. Differential diagnosis requires a thorough physical examination, medical history, imaging methods, and blood tests. Computed tomography (CT) has become the gold standard for right lower quadrant abdominal pain due to its high sensitivity in diagnosing AA [3].

Moreover, it has been proposed to use inflammatory biomarkers such as white blood cell (WBC) count, C-reactive protein (CRP), bilirubin level, immature granulocyte ratio, neutrophil-to-lymphocyte ratio (NLR), and platelet-to-lymphocyte ratio (PLR) to enable early diagnosis of AA and reduce the rate of misdiagnosis [4,5]. In addition, a study conducted on children revealed that MLR could be used to diagnose AA [6]. However, a single laboratory marker with a 100% diagnostic value to differentiate AA from other reasons for abdominal pain is yet to be available. Therefore, to provide the right treatment as soon as possible, the search for the most reliable, inexpensive, easy-to-implement, noninvasive, and easily accessible biomarkers continues.

As far as we know, there is no clinical research on the diagnostic value of the monocyte-to-lymphocyte ratio (MLR), red cell distribution width (RDW) to lymphocyte ratio (RLR), and systemic immune inflammation index (SII) in differentiating patients with and without AA in the adult patient group. Therefore, we hypothesized that MLR, RLR, and SII might serve the diagnostic process of AA in adult patients. This study aims to compare them with other biomarkers in distinguishing patients with and without AA.

## Materials and methods

Study design and settings

**Figure 1:**
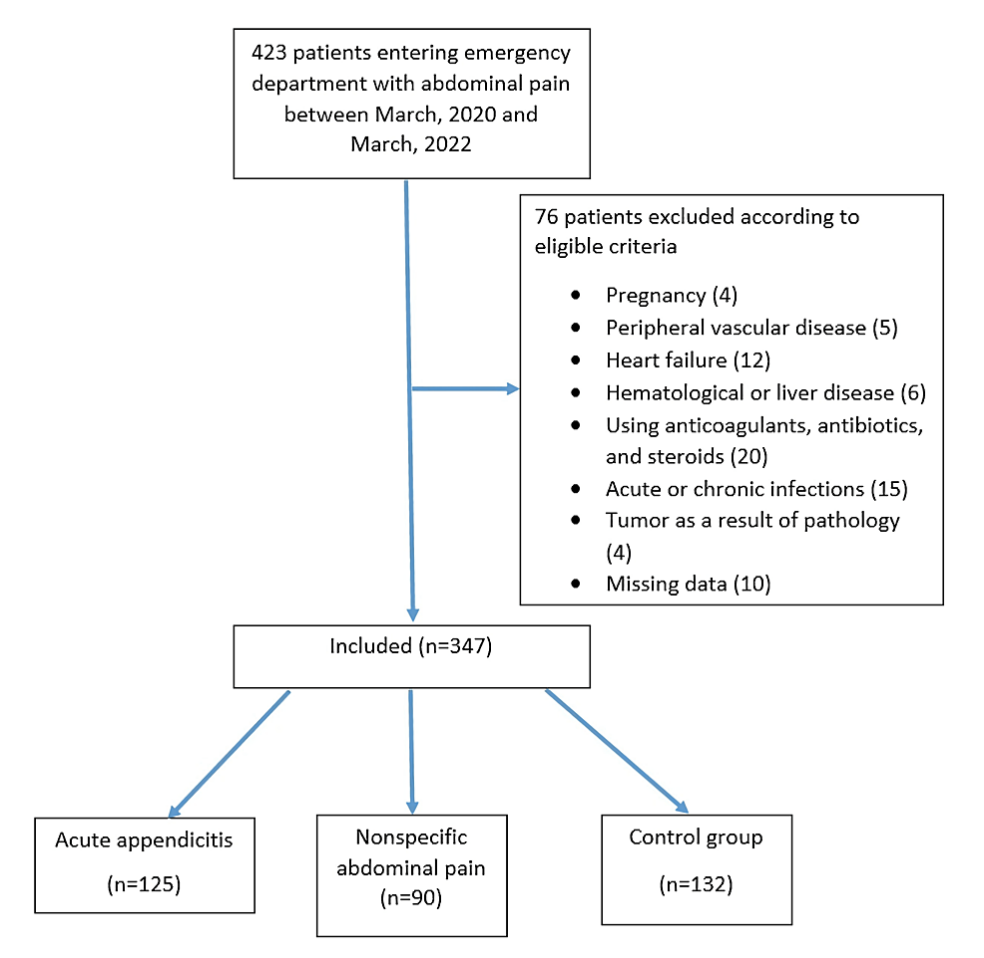
The patient's flowchart. Figure credits: Nafis Vural

According to the cross-sectional retrospective study, a total of 347 patients (AA 125, nonspecific abdominal pain [NAP] 90, and control group 132) were included in the study, using the inclusion and exclusion criteria among the patients who attended the emergency department with right lower abdominal pain between March 18, 2020, and March 18, 2022 (Figure 1). The study received approval, and the requirement for informed consent was waived by the Ethics Committee of Necmettin Erbakan University (ethics committee decision number 2022/3702 dated March 18, 2022). The research was carried out in accordance with the Declaration of Helsinki.

Power analysis

The main outcome variable, the NLR value, was used to determine the reliability assessment (post-study power) of the number of patients included in the groups. While NLR was 7.54 ± 5.26 in AA patients, it was 3.75 ± 4.2 in NAP patients. According to the difference in NLR levels between the independent group averages, the post-study power was 99%. According to the difference in the secondary outcome variables MLR, RLR, and SII, the post-study power was above 80%.

Study protocol

Patients aged 18 years or more were separated into AA, NAP, and the control group. The first group included patients who had an appendectomy with confirmed histopathological appendix inflammation. Patients who were not so confirmed were ruled out. The second group comprised patients who were admitted for clinical observation for suspected appendicitis but recovered completely without the need for surgical procedures. The third group consisted of patients who were seen in the emergency department with noninflammatory abdominal pain (renal colic, inguinal hernia, umbilical hernia, etc.). Demographic data of the groups, laboratory findings, surgical operation notes, pathology reports, and abdominal CT scan results at initial admittance to the emergency department were recorded. Patients who were pregnant; had the peripheral vascular disease; had suffered heart failure; had hematological or liver disease; were using anticoagulants, antibiotics, and steroids; had other acute or chronic infections; or had a pathology result revealing a tumor or whose records could not be accessed were all excluded. Patients who had an incidental appendectomy as part of another procedure were also excluded.

Laboratory analysis

Under aseptic conditions, 5 mL of blood was collected for a complete blood count (CBC) in an EDTA tube (BD Vacutainer, Becton Dickinson Diagnostics, Breda, The Netherlands) immediately after the admission of the patient. Within 30 minutes, blood samples were analyzed. The CBC was measured with an automated hematological analyzer (Sysmex Corporation, Kobe, Japan). The fluorescence flow cytometry method was used to differentiate the white cell population. Hematological parameters such as WBC, hemoglobin (Hgb), hematocrit (HTC), neutrophil count (NEU),lymphocyte (LYM)**,** monocyte (MON), platelet (PLT), RDW, NLR, MLR, RLR, and SII values were recorded. SII was calculated by multiplying PLT count and NLR [7]. In addition, CRP values were recorded. Mindray Chemistry Analyzer calculated these values (BS-2000M, Shenzhen, China).

Statistical analysis

Parametric tests were used without the normality test due to the compatibility of the central limit theorem [8]. In the analysis of the data, the mean and standard deviation and minimum and maximum values of the features were used while performing the statistics of continuous data. Categorical variables were defined using frequency and percentage values. One-way analysis of variance (ANOVA) test statistics were used to compare AA, NAP, and control group means. If ANOVA detected differences, they were evaluated with Tukey statistics as a post hoc test. Chi-square test statistics assessed the relationship between AA, NAP, control group, and sex. The cutoff in diagnostic value measurements was determined using the receiver operating characteristic (ROC) analysis. Statistical significance was determined by the statistics of sensitivity, specificity, positive predictive value (PPV), and negative predictive value (NPV). An AUC of 0.5 to 0.6 was interpreted as poor, 0.6 to 0.7 as fair, 0.7 to 0.8 as acceptable, 0.8 to 0.9 as excellent, and >0.9 as outstanding. Comparison of ROC curves for CRP, WBC, NEU, NLR, MLR, and SII were evaluated with a pairwise comparison of ROC curves and a 95% confidence interval. The level of statistical significance of the data is considered *P *< 0.05. New York software (e-picos, New York, NY, USA, www.e-picos.com) and the MedCalc statistical package program (MedCalc Software Ltd., Ostend, Belgium) were used for data evaluation and post-study power analysis.

## Results

A total of 347 patients (AA 125, NAP 90, and control 132) were included. The average and standard deviation values of age, sex, and biomarkers are shown in Tables 1-2. There is a significant relationship between the study groups and sex (*P *< 0.05). About 66.4% of the AA patients were male, 63% of the NAP patient group were female, and 50.8% of the patients in the control group were female. There was a significant difference between the group means of HGB, HTC, CRP, WBC, RDW, NEU, LYM, MON, NLR, MLR, SII, and RLR values (*P *< 0.05; Tables 1-2).

**Table 1:** Comparison of basic and laboratory characteristics of AA, NAP, and CG. *ANOVA. **Post hoc Tukey. AA, acute appendicitis; ANOVA, analysis of variance; CG, control group; NAP, nonspecific abdominal pain; SD, standard deviation; CRP, C-reactive protein; WBC, white blood cells; PLT, platelets; Hgb, hemoglobin; HCT, hematocrit; RDW, red cell distribution width; NEU, neutrophil; LYM, lymphocyte; MON, monocyte; NLR, neutrophil-to-lymphocyte ratio; RLR, RDW-to-lymphocyte ratio; MLR, monocyte-to-lymphocyte ratio; SII, systemic immune inflammation index

Features	Total (*N* = 347)	AA (*n* = 125)	NAP (*n* = 90)	CG (*n* = 132)	*P*-value^*^	*P*-value^**^		
	x̄ ± SD	x̄ ± SD	x̄ ± SD	x̄ ± SD		AA-NAP	AA-CG	NAP-CG
Age	40.2 ± 15.1	34.6 ± 14	41.1 ± 14.9	44.8 ± 14.6	<0.001	0.004	<0.001	0.15
CRP (mg/L)	24.71 ± 20.24	55.47 ± 25.45	12.11 ± 8.64	4.16 ± 3.72	<0.001	<0.001	<0.001	0.008
WBC (10^3 ^mcL)	10.91 ± 3.94	14.54 ± 3.59	9.45 ± 2.33	8.44 ± 2.22	<0.001	<0.001	<0.001	0.03
PLT (10^3 ^mcL)	267.06 ± 67.87	260.26 ± 61.38	276.54 ± 73.29	267.04 ± 69.62	0.22	-		
Hgb (g/L)	14.44 ± 1.76	14.84 ± 1.86	14.12 ± 1.52	14.27 ± 1.75	0.005	0.008	0.03	0.79
HTC (%)	42.29 ± 4.69	43.28 ± 4.91	41.37 ± 4.31	41.99 ± 4.59	0.008	0.008	0.07	0.58
RDW (fL)	13.25 ± 1.01	13.93 ± 0.93	13.23 ± 0.75	12.62 ± 0.79	<0.001	<0.001	<0.001	<0.001
NEU (10^3 ^mcL)	7.77 ± 4.08	11.07 ± 4.12	6.51 ± 3.133	5.45 ± 2.14	<0.001	<0.001	<0.001	0.02
LYM (10^3 ^mcL)	2.16 ± 0.91	1.76 ± 0.66	2.37 ± 1.01	2.39 ± 0.89	<0.001	<0.001	<0.001	0.98
MON (10^3 ^mcL)	0.74 ± 0.32	0.93 ± 0.29	0.68 ± 0.38	0.61 ± 0.22	<0.001	<0.001	<0.001	0.21
NLR	4.69 ± 3.48	7.54 ± 5.26	3.75 ± 4.2	2.64 ± 1.54	<0.001	<0.001	<0.001	0.09
RLR	7.53 ± 4.39	9.26 ± 4.5	7.09 ± 5.26	6.19 ± 2.87	<0.001	0.001	<0.001	0.26
MLR	0.39 ± 0.22	0.58 ± 0.23	0.32 ± 0.17	0.28 ± 0.12	<0.001	<0.001	<0.001	0.22
SII (PLT × NLR)	1215.71 ± 1073.33	1936.43 ± 1276.06	950.69 ±853.09	713.89 ± 457.58	<0.001	<0.001	<0.001	0.15

**Table 2:** Comparison of sex characteristics of AA, NAP, and CG (chi-square test). *Chi-square (*P* < 0.05 significance). AA, acute appendicitis; CG, control group; NAP, nonspecific abdominal pain

Features	Total (*N* = 347), n (%)	AA (*n* = 125), *n* (%)	NAP (*n* = 90), *n* (%)	CG (*n* = 132), *n* (%)	*P*-value^*^
Sex					
Female	166 (47.8)	42 (33.6)	57 (63.3)	67 (50.8)	<0.001
Male	181 (52.2)	83 (66.4)	33 (36.7)	65 (49.2)	

**Figure 2:**
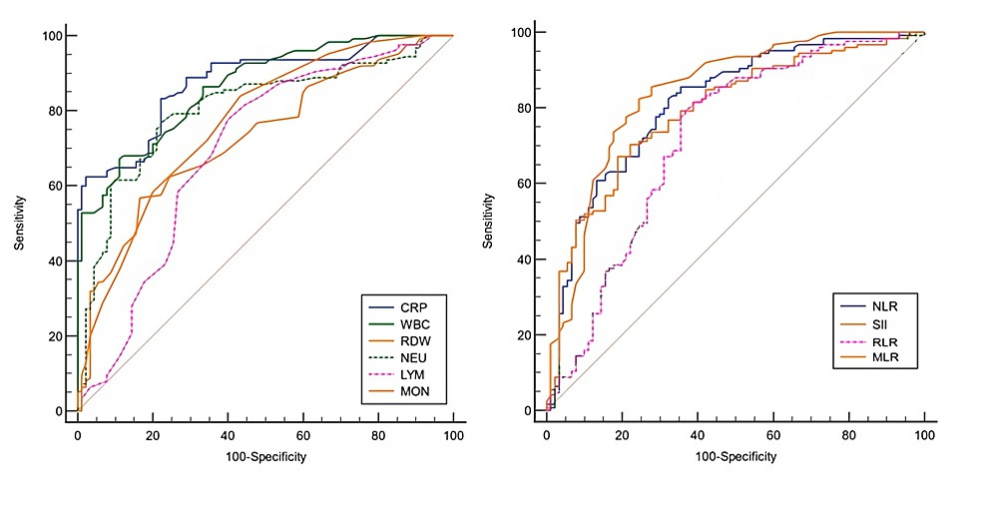
ROC curve of biomarkers for the diagnosis of acute appendicitis. CRP, C-reactive protein; WBC, white blood cells; RDW, red cell distribution width; NEU, neutrophil; LYM, lymphocyte; MON, monocyte; NLR, neutrophil-to-lymphocyte ratio; SII, systemic immune inflammation index; RLR, RDW-to-lymphocyte ratio; MLR, monocyte-to-lymphocyte ratio; ROC, receiver operating characteristic

The diagnostic accuracy of biomarkers important for the differential diagnosis of AA and NAP in ROC analysis is shown in detail in Table 3 (Figure 2). CRP, WBC, NEU, NLR, MLR, and SII were found to have excellent diagnostic power in AA detection (AUC 0.80-0.88). Results further indicated that RDW, LYM, MON, and RLR were of acceptable diagnostic power (AUC 0.70-0.77).

**Table 3:** Diagnostic accuracy of inflammatory parameters for differentiation of AA from NAP. AA, acute appendicitis; NAP, nonspecific abdominal pain; AUC, area under curve; PPV, positive predictive value; NPV, negative predictive value; CI, confidence interval; CRP, C-reactive protein; WBC, white blood cells; RDW, red cell distribution width; NEU, neutrophil; LYM, lymphocyte; MON, monocyte; NLR, neutrophil-to-lymphocyte ratio; RLR, RDW-to-lymphocyte ratio; MLR, monocyte-to-lymphocyte ratio; SII, systemic immune inflammation index

AA (125) NAP (90)	AUC	Cutoff	Sensitivity (%)	Specificity (%)	AUC (95% CI)	P-value	PPV (%)	NPV (%)
CRP (mg/L)	0.88	>19.2	83.2	77.78	0.83-0.92	<0.001	83.9	76.9
WBC (10^3 ^mcL)	0.87	>12.2	67.2	88.89	0.81-0.91	<0.001	89.4	66.1
RDW (fL)	0.73	>13.8	56.8	83.33	0.67-0.79	<0.001	82.6	58.1
NEU (10^3^ mcL)	0.81	>82	76.4	77.8	0.75-0.86	<0.001	82.8	70.7
LYM (10^3 ^mcL)	0.70	≤2.1	77.6	60	0.63-0.76	<0.001	72.9	65.9
MON (10^3 ^mcL)	0.77	>0.6	84	56.7	0.71-0.82	<0.001	72.9	71.8
NLR	0.81	>3.67	82.4	67.78	0.75-0.86	<0.001	78.1	73.5
RLR	0.72	>6.27	81.6	61.1	0.65-0.78	<0.001	74.5	70.5
MLR	0.84	>0.33	82.4	75.6	0.78-0.89	<0.001	82.4	75.6
SII	0.80	>1218.97	67.2	81.1	0.74-0.85	<0.001	83.2	64.1

**Figure 3:**
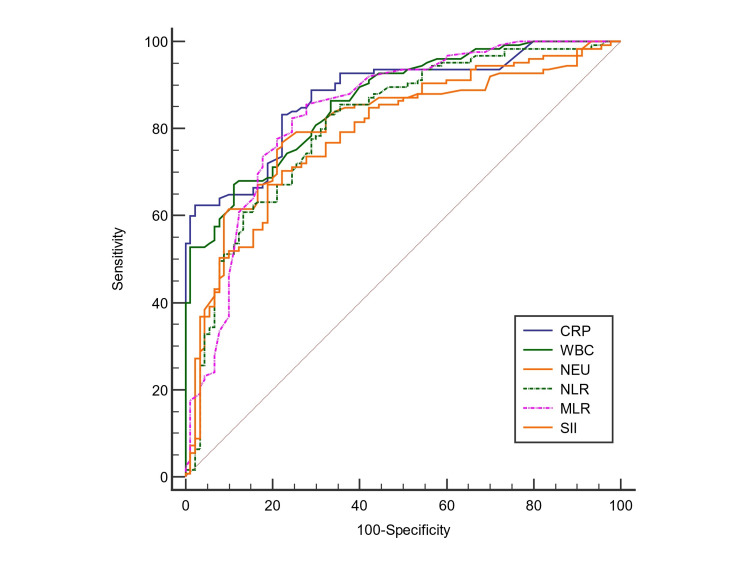
Pairwise comparison of ROC curves for CRP, WBC, NEU, NLR, MLR, and SII. CRP, C-reactive protein; WBC, white blood cells; NEU, neutrophil; NLR, neutrophil-to-lymphocyte ratio; MLR, monocyte-to-lymphocyte ratio; SII, systemic immune inflammation index; ROC, receiver operating characteristic

When the similarities of CRP, WBC, NEU, NLR, MLR, and SII in the diagnosis of AA were evaluated by the comparison of ROC curves, significant differences were found between CRP and NEU, CRP and SII, WBC and NEU, and WBC and SII (*P *< 0.05). However, there was no difference between the AUC of other diagnostic parameters (*P *> 0.05). Therefore, we found that parameters that are not statistically different can be used interchangeably when diagnosing AA (Table 4, Figure 3).

**Table 4:** Pairwise comparison of ROC curves: difference between areas, 95% confidence interval, and P-values. CRP, C-reactive protein; WBC, white blood cells; NEU, neutrophil; NLR, neutrophil-to-lymphocyte ratio; MLR, monocyte-to-lymphocyte ratio; SII, systemic immune inflammation index; ROC, receiver operating characteristic

	Difference between areas	95% confidence interval	*P*-value
CRP-WBC	0.01	0.001-0.068	0.65
CRP-NEU	0.07	0.002-0.14	0.04
CRP-NLR	0.06	0.006-0.14	0.07
CRP-MLR	0.04	0.03-0.11	0.28
CRP-SII	0.09	0.01-0.16	0.02
WBC-NEU	0.06	0.01-0.11	0.01
WBC-NLR	0.05	0.008-0.11	0.09
WBC-MLR	0.02	0.04-0.09	0.46
WBC-SII	0.07	0.02-0.13	0.01
NEU-NLR	0.006	0.003-0.05	0.76
NEU-MLR	0.04	0.03-0.1	0.31
NEU-SII	0.02	0.02-0.06	0.46
NLR-MLR	0.03	0.02-0.08	0.29
NLR-SII	0.02	0.003-0.05	0.09
MLR-SII	0.05	0.008-0.11	0.1

## Discussion

Important new findings of this study are that RLR, SII, and MLR are valuable parameters in differentiating patients with and without appendicitis in adult patients presenting to the emergency department with abdominal pain.

Acute abdomen accounts for 7%-10% of all emergency department admissions. AA is the fourth most frequent cause of acute abdomen pain with a rate of 3.8% [1]. Emergency physicians are always looking for a noninvasive, reliable tool to detect life-threatening conditions in patients. As AA causes a systemic inflammatory response, simple, easy-to-apply, and inexpensive inflammatory biomarkers are still used in differential diagnosis [9]. However, none of these biomarkers have sufficient predictive value to exclude AA. One of the limitations of these markers is their dependence on the clinical stage of the disease [10]. Another limitation is that AA symptoms can be mimicked by a variety of nonsurgical conditions, the majority of which are related to inflammation and/or infection. Limitations lead to delays in diagnosing and emerging complications [11]. This situation has forced scientists to research new biomarkers.

WBC count is the most commonly used laboratory test in diagnosing AA. Yang et al. discovered that leukocytosis was the earliest laboratory test to show AA and that the sensitivity and specificity of leukocytosis were 87.2% and 33.1%, respectively [12]. CRP, a positive acute-phase protein, rises in inflammatory processes. Xharra et al. found that CRP had a positive predictive value of 94.7%, specificity of 72%, and sensitivity of 85.1% for AA [13]. Muller et al. calculated CRP sensitivity at 61% and specificity at 58%, with a 95% confidence interval in their study for AA diagnostic accuracy [14]. In this study, while the sensitivity and specificity of CRP were similar to those reported in the literature, there were minor differences in WBC count.

NLR provides information about two separate inflammatory pathways, making it a potential marker to predict AA and its severity. The NEU count highlights the active and ongoing inflammatory state, while the LYM count highlights the regulatory pathway [15]. According to Hajibandeh et al., NLR is a promising marker that can predict the diagnosis and severity of AA with a cutoff value of 4.7, AUC of 0.96, sensitivity of 88.89%, and specificity of 90.91% [4]. Fatima et al. calculated the sensitivity of the NUE test as 88.03% and specificity as 92.86% in the diagnosis of AA (AUC 0.904) [16]. The AUC of NLR (0.81) was determined to be lower in our study than in the literature.

Monocytosis was used as an indicator for various inflammatory diseases [17]. Previous research has shown that patients affected by appendicitis, particularly those with gangrene, have notable lymphopenia [18]. Through these mechanisms, we can understand the reason for the rise of MLR in patients with appendicitis. Duman et al. showed that MLR with 0.47 cutoff, 75.98% sensitivity, 72.08% specificity, and 0.798 AUC is a biomarker that can be used to differentiate acute appendicitis and nonspecific abdominal pain in pediatric patients [19]. In our study, it was discovered that MLR could also be used in the diagnosis of AA in adult patients.

SII is a new inflammatory index that comprehensively reflects the host immune and inflammatory state balance [20]. A high SII score is associated with adverse outcomes in cancer patients, heart failure, and coronary artery disease [7]. It has even been proposed that SII is more useful than NLR and PLR alone in predicting inflammatory status and prognosis in a variety of clinical scenarios [21]. In light of the data of this study, it was seen that SII is also closely related to AA in adults.

RLR is another biomarker used in recent studies. Wu et al. demonstrated high sensitivity and specificity of RLR in predicting hepatic impairment in patients with the hepatitis E virus [22]. Meng et al. showed that RLR could determine the severity of primary biliary cirrhosis due to its high diagnostic specificity [23]. Furthermore, RLR was demonstrated to have acceptable diagnostic power in detecting acute appendicitis in pediatric patients [6]. According to the results of our study, we calculated that RLR could be used in the diagnosis of acute appendicitis with a cutoff of 6.27.

There were a few limitations to our research. This was a single-center retrospective study. We used laboratory results from the first application for analysis and did not include follow-up values. The time between the onset of symptoms and admission to the emergency department was unknown. Therefore, it is not known which part of the inflammatory process coincides with the time when the laboratory parameters are studied. As such, our findings cannot be generalized; however, they may be informative for future studies for more reliable and precise results.

## Conclusions

The diagnosis of AA still depends on many factors, including clinical evaluation, laboratory tests, and radiological imaging. Inflammatory biomarkers could help this process. MLR and SII, which are simple, inexpensive, and easily accessible parameters, may be recommended to be used together with other clinical findings in diagnosing AA in adult patients. RLR is acceptable but not superior.
